# Biomarkers for Duchenne muscular dystrophy: myonecrosis, inflammation and oxidative stress

**DOI:** 10.1242/dmm.043638

**Published:** 2020-02-03

**Authors:** Miranda D. Grounds, Jessica R. Terrill, Basma A. Al-Mshhdani, Marisa N. Duong, Hannah G. Radley-Crabb, Peter G. Arthur

**Affiliations:** 1 School of Human Sciences, the University of Western Australia, Perth, WA 6009, Australia; 2 School of Molecular Sciences, the University of Western Australia, Perth, WA 6009, Australia; 3 School of Pharmacy and Biomedical Sciences, Curtin Health and Innovation Research Institute, Faculty of Health Sciences, Curtin University, Perth, WA 6102, Australia

**Keywords:** DMD, Dystrophic mice, Rats, Dogs, Biomarkers, Blood, Urine, Muscle necrosis, Inflammation, Neutrophils, Oxidative stress

## Abstract

Duchenne muscular dystrophy (DMD) is a lethal, X-linked disease that causes severe loss of muscle mass and function in young children. Promising therapies for DMD are being developed, but the long lead times required when using clinical outcome measures are hindering progress. This progress would be facilitated by robust molecular biomarkers in biofluids, such as blood and urine, which could be used to monitor disease progression and severity, as well as to determine optimal drug dosing before a full clinical trial. Many candidate DMD biomarkers have been identified, but there have been few follow-up studies to validate them. This Review describes the promising biomarkers for dystrophic muscle that have been identified in muscle, mainly using animal models. We strongly focus on myonecrosis and the associated inflammation and oxidative stress in DMD muscle, as the lack of dystrophin causes repeated bouts of myonecrosis, which are the key events that initiate the resultant severe dystropathology. We discuss the early events of intrinsic myonecrosis, along with early regeneration in the context of histological and other measures that are used to quantify its incidence. Molecular biomarkers linked to the closely associated events of inflammation and oxidative damage are discussed, with a focus on research related to protein thiol oxidation and to neutrophils. We summarise data linked to myonecrosis in muscle, blood and urine of dystrophic animal species, and discuss the challenge of translating such biomarkers to the clinic for DMD patients, especially to enhance the success of clinical trials.

## Abstract (precis)

**Summary:** This Review discusses biomarkers in blood and urine linked to myonecrosis, inflammation and oxidative stress, to enhance development of therapies for DMD, and the challenges to be overcome for clinical translation.

## Introduction: pathophysiology of Duchenne muscular dystrophy

Developing robust biomarkers for a disease requires comprehensive information about the human condition and the associated animal models. Duchenne muscular dystrophy (DMD) is a lethal, X chromosome-linked muscle disease caused by mutations in the dystrophin (*DMD*) gene, which result in the loss or altered function of dystrophin protein. DMD affects about 1 in 3500-6000 boys worldwide, causing severe loss of muscle mass and function, with death often occurring in the late teens due to respiratory or cardiac failure (Bushby et al., 2010; Falzarano et al., 2015; Partridge, 2011). *DMD* is the largest gene in the human genome and encodes at least seven distinct proteins; one of which, the dystrophin isoform Dp427, is found in skeletal and cardiac muscle. All dystrophin isoforms bind to a dystroglycan complex (DGC) in the cell membrane (Waite et al., 2012). In skeletal muscles, dystrophin is located beneath the sarcolemma (Box 1, Glossary) and links the actin cytoskeleton and the specialised contractile proteins in the sarcoplasm (Box 1) to the transmembrane DGC that spans the sarcolemma to connect with laminin and a network of extracellular matrix (ECM) molecules, including collagens, to transfer the contractile muscle force and move parts of the skeleton. Dystrophin is enriched at the costameres and myotendinous junctions (MTJs; Box 1) where force is transmitted across the cell membrane (Ridge et al., 1994; Zhao et al., 1992). Dystrophin is also involved in various signalling pathways (Allen et al., 2016).

**Box 1. Glossary**

**Costamere**. The structural-functional component of striated myofibres that links the sarcomere to the cell membrane.

**Creatine kinase (CK)**. Enzyme expressed in muscle and other tissues that catalyses the conversion of creatine to phosphocreatine and adenosine diphosphate.

**Myoglobin**. Iron- and oxygen-binding protein found in myofibres; particularly abundant in slow muscles, which are better suited to derive their energy by oxidative phosphorylation.

**Myotendinous junctions (MTJs)**. Site of connection between tendon and muscle.

**Neuromuscular junctions (NMJs)**. Site of the transmission of action potential from nerve to muscle.

**Nitric oxidase synthase (NOS)**. Enzyme catalysing the production of nitric oxide.

**Sarcolemma**. Cell membrane of a striated myofibre.

**Sarcoplasm**. Cytoplasm of a striated myofibre.

**6-min walk test**. A clinical test protocol that measures the total distance DMD patients are able to walk in 6 min.

**Xanthine oxidase**. Enzyme that catalyses the oxidation of hypoxanthine to xanthine and can further catalyse the oxidation of xanthine to uric acid.

**Fig. 1.**
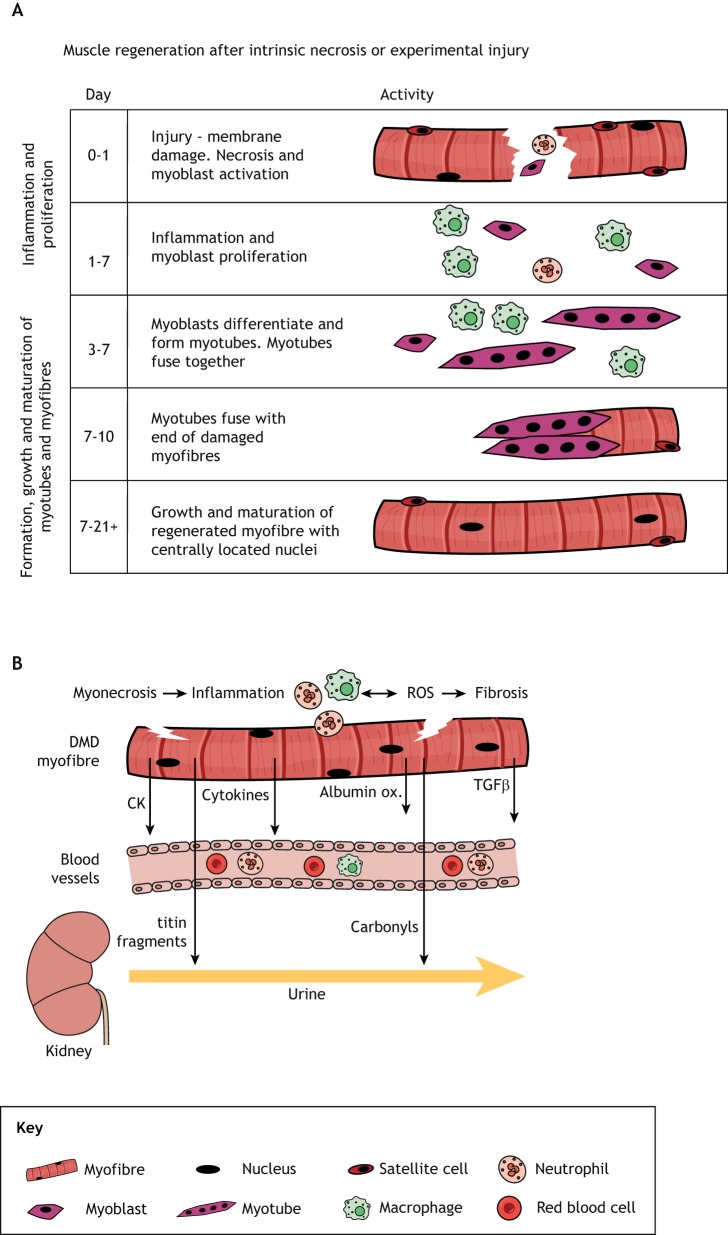
**Necrosis of dystrophic skeletal muscle and associated cellular events.** (A) Timeline of events resulting from experimental necrosis of normal muscle. This diagram indicates the timing of the main events associated with regeneration of normal muscle after a single bout of myonecrosis upon experimental injury (Grounds, 2014; Radley-Crabb et al., 2014). A similar sequence of events occurs in dystrophic muscle after intrinsic myonecrosis, although the environment is progressively altered by repeated bouts of damage, with disturbed inflammatory cell populations and increasing fibrosis that can impair myogenesis and regeneration. (B) Simple diagram to indicate biomarkers in dystrophic muscle associated with the key events of myonecrosis. Some biomarkers are present only in muscle, whereas others can be detected in blood or urine (see Table 1 and text for details). Albumin ox., oxidised albumin; CK, creatine kinase; ROS, reactive oxygen species.

Mutations in *DMD* causing a lack of functional dystrophin result in a fragile sarcolemma that is susceptible to damage after skeletal muscle contraction, leading to intrinsic myofibre necrosis (or myonecrosis). Necrosis is closely associated with increased inflammation and oxidative stress (Tidball et al., 2018), and leads to subsequent regenerative myogenesis (Fig. 1). Repeated bouts of myonecrosis also cause increased fibrosis over time (Allen et al., 2016; Biggar, 2006; Bushby et al., 2010; Emery, 2002; Falzarano et al., 2015; Grounds, 2008; Kharraz et al., 2014; Kim et al., 2013; Renjini et al., 2012). Intrinsic myonecrosis of skeletal muscles is central to the progressive dystropathology of DMD and appears to be exacerbated by growth, exercise and metabolism, associated with unmet high energy needs (Radley-Crabb et al., 2014). In addition, the progressively increasing fibrosis caused by repeated bouts of myonecrosis and inflammation impairs myogenesis and regeneration of DMD muscles, with resultant severe loss of muscle tissues.

**Table 1.**
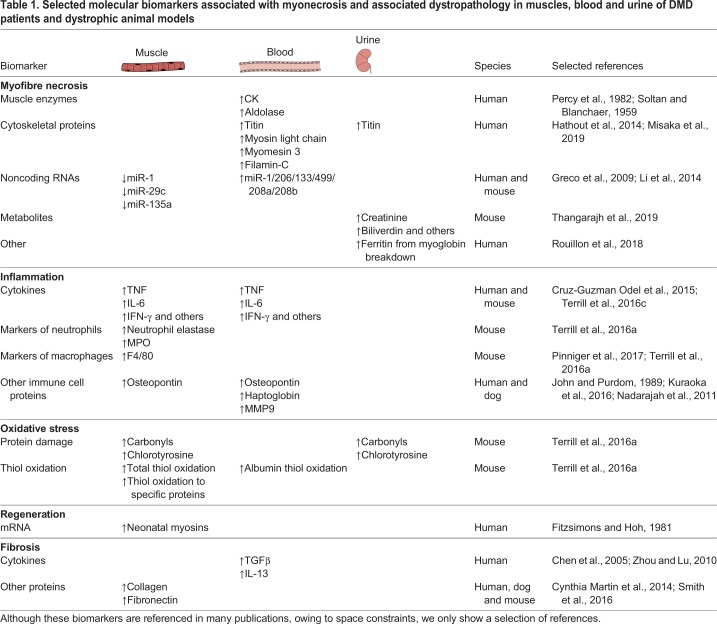
**Selected molecular biomarkers associated with myonecrosis and associated dystropathology in muscles, blood and urine of DMD patients and dystrophic animal models**

Bouts of intrinsic myonecrosis in DMD can also directly damage neuromuscular junctions (NMJs; Box 1). The adverse progressive changes in NMJs, which indicate denervation, are widely reported in dystrophic muscles of rodent and dog models of DMD (Haddix et al., 2018). These altered NMJs affect the associated dystrophic nerve over time, with consequent increased levels of S100 and Tau5 proteins seen by 13 months of age in sciatic nerves of *mdx* mice (Gordish-Dressman et al., 2018). Such neuronal changes indicate progressive irreversible denervation, often associated with neurodegeneration (Krishnan et al., 2016), that is likely to become pronounced over many years or decades and contribute to the loss of muscle function in DMD patients. These preclinical neuronal changes could prove useful as a biomarker for the long-term consequences of repeated intrinsic myonecrosis in animal studies.

A key aim for DMD therapies is to prevent myonecrosis and to directly stabilise the myofibres, ideally by replacing the non-functional dystrophin using various gene delivery or molecular strategies, with recent promising progress (Verhaart and Aartsma-Rus, 2019). In parallel, there is interest in optimising therapies to either prevent or reduce myonecrosis, or target the associated events of inflammation, oxidative stress, failed regeneration, fibrosis and neurodegeneration to try and maintain muscle integrity and function.

One of the many challenges in developing a therapy for DMD is the long treatment times required before a meaningful functional clinical outcome can be obtained. For example, to assess therapeutic benefits with the 6-min walk test (Box 1), patients need to receive treatment for 6-9 months. Consequently, clinical trials are expensive, resource-intensive and time consuming, and require considerable commitment from families. Another issue is the need to determine a suitable clinical dosing regimen for young DMD boys, as toxicology studies and optimal doses are often determined preclinically in adult animal models that do not necessarily translate to humans (Reagan-Shaw et al., 2008), and repurposed drugs that have been used previously for ‘normal’ adult humans may have a very different impact in growing children (Barker et al., 2018), especially those with severe muscle damage. Thus, it is highly desirable to have suitable rapidly responsive and accessible molecular biomarkers of DMD to help determine the best route, frequency and dose of treatment before undertaking a full clinical trial.

In addition, robust biomarkers would be clinically valuable in helping to assess the capacity of a therapy to specifically reduce myonecrosis and hence disease severity over time. Thus, the central aim of this article is to discuss molecular biomarkers in muscle, blood and urine as reliable and mechanistically relevant readouts of the extent of myonecrosis in DMD. Necrosis is the central process that causes the progressive pathogenesis of DMD, and thus biomarkers that can quantify its extent have the potential to acutely track disease progression. We therefore focus our discussion on the events of myonecrosis and of the closely associated inflammation and oxidative stress. Biomarkers of the consequent and delayed occurrence of fibrosis and fatty replacement of muscles, along with neurodegeneration, fall outside the scope of this Review. The progressive changes in tissue composition, which measure the severity of dystropathology over time, can be monitored by magnetic resonance imaging (MRI) in humans and animal models of DMD (Szigyarto and Spitali, 2018). Although MRI is increasingly being used as a powerful tool for measuring outcome without the need for muscle biopsy, its repeated use has limitations, including expensive equipment and expertise, high cost per measurement and inconvenience for the patient due to the immobilisation and time required for repeated measurements (Szigyarto and Spitali, 2018). For therapies using a drug that targets a specific molecular signalling pathway, it is clearly desirable to monitor the predicted changes in proteins or RNAs within that pathway to demonstrate drug target engagement and efficacy. However, such drug-specific pharmacodynamic biomarkers fall outside the scope of the present discussion.

Diverse dystrophic animal models are used to study DMD, ranging from the classic *mdx* mouse (Coulton et al., 1988; Partridge, 2013) and the important larger dystrophic dog models such as golden retriever muscular dystrophy (GRMD) with more severe disease manifestation (reviewed by Kornegay, 2017), to dystrophic *Dmd^md^* rats (Larcher et al., 2014), rabbits (Sui et al., 2018), pigs, cats, zebrafish and fruit flies (reviewed by Wells, 2018). Although human blood and urine samples can be fairly easily obtained for analyses, muscle biopsy is highly invasive and undesirable for DMD patients. Thus, data from diverse tissue samples of animal models provide the basis for much of the following discussion.

## Overview of molecular biomarkers, especially for myonecrosis and associated events

Many biomarkers of potential interest for DMD that reflect the primary feature of myonecrosis, associated inflammation and oxidative stress, as well as secondary disturbances such as fibrosis, have been identified in muscle tissue and biofluid samples, mainly blood and urine (Table 1), but very few are in routine clinical and experimental use. Some candidate DMD biomarkers have been identified in several studies and species, and their responsiveness to therapies demonstrated, whereas others may have been identified in a single study or show wide variation between individuals and between studies (Table 1 and discussed below). For example, one classic widely used clinical blood (plasma) biomarker for DMD is the enzyme creatine kinase (CK; Box 1), which is elevated in patients and in rodent and dog DMD models, but can be highly variable (reviewed by Dowling et al., 2019; Hathout et al., 2014; Szigyarto and Spitali, 2018). Nevertheless, increased CK levels, specifically the MM muscle form measured by immunoassay in dried bloodspots, are now being used for newborn DMD screening (Moat et al., 2017).

More recently, extensive proteomic, RNA and metabolite analyses have been carried out in animal models and patients, as discussed in a number of excellent reviews on potential biomarkers for muscle, blood and urine (Aartsma-Rus et al., 2018; Aartsma-Rus and Spitali, 2015; Dowling et al., 2019; Hathout et al., 2014, 2016; Lourbakos et al., 2017; Parolo et al., 2018; Szigyarto and Spitali, 2018; Thangarajh et al., 2019). A large-scale proteomic approach to identify serum biomarkers associated with pathophysiological change over time (Spitali et al., 2018) concluded that ∼33 proteins were bona fide biomarkers as they were able to discriminate between DMD patients and healthy controls in all cohorts, with a concordant directional change towards either a consistent increase or decrease in patients.

## Quantification of necrosis in dystrophic muscles

Identifying biomarkers in body fluids that reflect the primary events of myonecrosis and the closely associated oxidative stress and inflammation, which usually result in regeneration, requires an accurate assessment of necrosis in muscles. This section first discusses the factors contributing to the onset of myonecrosis and the techniques to quantify myonecrosis and subsequent early regeneration (Fig. 1A), followed by molecules that can be measured in biofluids.

### Onset and exacerbation of myonecrosis

Although the precise events that initiate the intrinsic sarcolemma damage and consequent myonecrosis in DMD are not fully understood, small membrane breaks, increased intracellular calcium, inflammation and oxidative stress are strongly implicated and are closely linked (reviewed by Allen et al., 2016; Arthur et al., 2008). As interventions that target any of these can prevent the transition from sarcolemmal damage and leakiness to irreversible necrosis, it can be difficult to ascertain precisely what the critical initiating event is. Electron microscopy studies of DMD muscles identified overcontraction of myofibres as an early mechanical event, along with leakiness and small physical gaps in the sarcolemma, that support the notion of mechanical events initiating the catastrophic myonecrosis cascade (Cullen and Fulthorpe, 1975; Schmalbruch, 1975). However, other data support calcium influx dysregulation as the initiating event (reviewed by Allen et al., 2016; Kornegay, 2017). Although the molecular events leading to it are not fully understood, myonecrosis causes histological changes that can be quantified.

### Histological quantification of recent myonecrosis and early regeneration

**Fig. 2.**
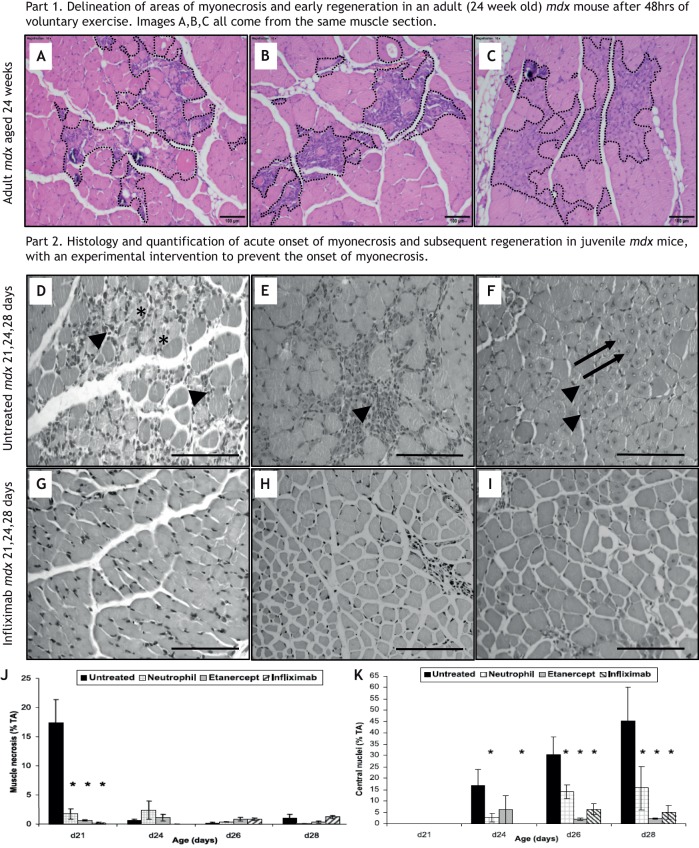
**Quantification of myonecrosis and subsequent regeneration in muscle tissue sections of young *mdx* mice at acute onset of myonecrosis (∼21 days postnatal).** (A-C) H&E-stained transverse sections of paraffin-embedded tibialis anterior (TA) muscles from young *mdx* mice aged 21 to 28 days (adapted from Hodgetts et al., 2006). This image is not published under the terms of the CC-BY license of this article. For permission to reuse, please see Hodgetts et al. (2006). (D-K) Untreated young *mdx* mice (D,E,F) and young *mdx* mice treated with a TNF blocking antibody, infliximab (also known as Remicade) (G,H,I), injected intraperitoneally from 7 days of age once a week, with mice sampled at 21, 24 and 28 days of age. In control untreated *mdx* muscles, areas of new myonecrosis are present with fragmented sarcoplasm (asterisks) and some inflammatory cells (arrowheads) at 21 days (D), with foci of recent myonecrosis and early regeneration evident by pronounced inflammation and young myogenic cells (arrowhead) present by 24 days (E), and advanced regeneration with small plump myotubes (arrowheads) with central myonuclei (arrows) conspicuous by 28 days (F). (Note that only D and E would be classified as representing recent myonecrosis for quantification purposes.) This acute onset of myonecrosis and subsequent events are not evident in the treated mice (G,H,I), as clearly shown by the quantification data in J and K. Quantification is shown for the proportion (%) of muscle tissue occupied by myofibre necrosis (J) and myoblasts/myotubes with central myonuclei (K; as a marker of regeneration), for untreated *mdx* mice sampled at days 21-28, compared with three groups of *mdx* mice that received TNF-reducing treatment to prevent the acute onset of myonecrosis: neutrophil depletion, soluble receptors to TNF (etanercept, also known as Enbrel) or inflixamab antibody to TNF (for details see Hodgetts et al., 2006). *n*=6 mice per group. *P<0.05 between untreated control *mdx* mice and treatment group at a specific time point (two-way ANOVA). Data are mean±s.e.m. Scale bars: 100 µm.

Skeletal myonecrosis is routinely assessed in tissue sections by simple Haematoxylin and Eosin (H&E) histological staining of transverse muscle sections as described in the Standard Operating Procedures (SOPs) on the TreatNMD website (see TreatNMD, DMD_M.1.2.007; http://www.treat-nmd.eu/downloads/file/sops/dmd/MDX/DMD_M.1.2.007.pdf). As there is sometimes confusion about the specific histological criteria to identify and quantify necrosis of myofibres, we discuss them in more detail here and in Fig. 2, in which myonecrosis is shown to occur upon acute experimental injury (day 0).

Myonecrosis is a form of cell death associated with the presence of inflammatory cells and is identified by light microscopy as paler staining and fragmentation of the sarcoplasm. Although fragmentation alone can be sufficient, the presence of inflammatory cells within myofibres (Figs 1 and 2) confirms recent myonecrosis (Coulton et al., 1988). The precise molecular mechanisms involved in the physical breakdown of myofibre structure are not clear and could involve necroptosis or other cellular events (Morgan et al., 2018). When these features of myonecrosis are combined with evidence of early myogenesis and regeneration, such as basophilic myoblasts and small myotubes formed by day 4 (Fig. 2B,C,E), they identify the incidence of recent active focal necrosis with early regeneration. The timing of these events is similar to the exercise-induced myonecrosis in adult *mdx* mice (Radley-Crabb et al., 2012). Transverse sections of *mdx* muscles often show a small cluster of several adjacent myofibres undergoing necrosis, whereas longitudinal views reveal that overcontraction and necrosis often affect only a short segment of the dystrophic myofibre, called focal or segmental necrosis (Blaveri et al., 1999; Coulton et al., 1988; Cullen and Fulthorpe, 1975). Analyses of many *mdx* mice aged 12 weeks demonstrate that individual animals can exhibit high biological variation in the incidence of myonecrosis (Radley-Crabb et al., 2011).

### Embryonic or neonatal myosin isoforms

Immunostaining of embryonic or neonatal myosins is a classic technique used to identify newly formed, i.e. regenerating, myotubes and myofibres in mature muscles. However, this can also be subject to ambiguity. Although new myotubes initially express embryonic myosin, recapitulating embryogenesis, this is replaced by mature myosin isoforms during maturation. Conversely, denervated myofibres re-express embryonic myosin. Thus, the precise reason for a myofibre expressing embryonic or neonatal myosin needs to be carefully considered (Grounds, 2014). This is especially difficult in clinical muscle biopsies and if the precise history is not known. This problem is exemplified by the situation in which experts initially classified small neonatal myosin-expressing myofibres in human muscle biopsies as ‘regenerating myofibres’, but subsequently considered that the majority of these were instead ‘mature, small-sized, and truly atrophic’, and thereafter used the term atrophic to refer to neonatal myosin-positive small-sized fibres (Fanin et al., 2014).

### Central myonuclei

Once a mouse muscle has completed a bout of regeneration after intrinsic damage or experimental injury, the myonuclei persist in a central position for many months instead of re-locating to the periphery in the normal sub-sarcolemmal position (Grounds, 2014). Therefore, the presence of centrally located myonuclei in a tissue section (Fig. 2F,K) is widely used to identify myofibres that have regenerated in the past. As persisting central myonuclei are the outcome of many bouts of myonecrosis at varying times (Coulton et al., 1988), this measure does not identify recent necrosis and early myogenesis/regeneration, but is instead a useful proxy of cumulative muscle regeneration (Grounds, 2014). Indeed, *mdx* mice experience acute myonecrosis at 3 weeks of age, and ∼80% of adult mice (12 weeks of age) have myofibres with centrally located nuclei that persist for many months (Haddix et al., 2018). Consequently, central myonuclei are of limited use in identifying any striking reduction in recent active myonecrosis and subsequent regeneration in adult *mdx* mice, after the acute phase of necrosis/regeneration has occurred. Instead, the incidence of myonecrosis is best measured by direct histological quantification (see above). Even very old *mdx* mice have an excellent capacity for new muscle formation in the limb muscles, so when a study states that a particular intervention has ‘improved muscle regeneration’, it is difficult to know what this actually means and requires critical consideration. Specifically, when this statement is based on an increased number of myofibres with central myonuclei, this might instead reflect more myonecrosis (Grounds, 2014).

### Labelling of blood components that enter leaky and necrotic myofibres

Disturbed integrity of the sarcolemma can result in various molecules from the blood and interstitial fluid leaking into myofibres, where they can be visualised. A classic example is albumin, which can be identified in tissue sections with antibodies to demonstrate myofibre permeability (Straub et al., 1997). In addition to such an intrinsic marker, various dyes and contrasting agents that bind to albumin are also very useful, although these have to be administered into the animal or patient before tissue analysis. Evans blue dye (EBD), which binds to albumin, is widely used in animal studies to label all leaky myofibres in frozen muscle tissue sections (Hamer et al., 2002) and in whole muscles (Straub et al., 1997). The use of contrasting agents in the blood for non-invasive *in vivo* MRI measurements is also useful for analyses of intact animals (Amthor et al., 2004). Although these markers enter into all leaky myofibres of the body, it is critical to emphasise that such leakiness may be transitory and is not always associated with myonecrosis (Hamer et al., 2002; Straub et al., 1997); care is therefore needed in interpretation. In addition, EBD studies confirm that sarcolemmal leakiness can vary significantly between individual mice and muscles (Straub et al., 1997). It should also be considered that albumin and other blood molecules may be more readily released from leaky blood vessels of dystrophic animals or DMD patients, as dystrophin is also expressed in vascular endothelial cells (Palladino et al., 2013) and DMD capillaries show disturbed morphology (Miike et al., 1987).

In conclusion, we consider that, for preclinical studies, the histological measurement of areas of initial myonecrosis with early regeneration on H&E-stained sections can be useful to identify recent bouts of necrosis (shown in Fig. 2 for juvenile *mdx* and adult *mdx* mice subjected to exercise). Use of neonatal myosin isoforms to identify newly formed myotubes and myofibres, which can indicate regeneration, can also be useful, but caution is needed owing to possible misinterpretation in human biopsies. Although quantification of myofibres with central myonuclei is useful as an overall cumulative measure of myofibres that have undergone necrosis and regeneration, this is difficult to employ to demonstrate any subsequent reduction in the incidence of myonecrosis after the initial acute damage has occurred. As a consequence, measuring central myonuclei has the potential to provide misleading information when attempting to relate the incidence of myonecrosis to biomarkers in biofluids.

### Molecules in biofluids as biomarkers of myonecrosis

Dystrophic muscles secrete molecules into blood, and some of these can also be excreted in urine, which allows for relatively easy collection and quantification of muscle-derived biomarkers. The disturbed integrity of the dystrophic sarcolemma and myonecrosis exacerbate leakage. Several muscle-derived proteins, metabolites, RNAs and other molecules in the blood have been widely studied using a range of technologies (reviewed by Szigyarto and Spitali, 2018) and include enzymes such as CK and aldolase in the blood, structural proteins associated with sarcomeric contraction such as myosin light chain 1/3, myomesin 3 and fragments of titin in urine, breakdown products of myoglobin (Box 1) with ferritin in urine, and intermediate filaments such as filamin C (see Table 1). For blood biomarkers, it is important to consider whether they are measured in serum or plasma, and the anti-clotting agent for plasma samples needs to be carefully selected as sample preparation can influence detection of specific biomarkers (Szigyarto and Spitali, 2018).

Over the last decade, there has been expanding interest in non-coding (nc)RNAs, which include micro (mi)RNAs, long non-coding (lnc)RNAs and many other forms. Although miRNAs have been most widely investigated, a recent study identified differences in expression of lncRNAs and transfer (t)RNAs between growing muscle of dystrophic *mdx* and normal mice (Butchart et al., 2018). As many ncRNAs have tissue-specific expression patterns, are released into the blood stream and are stable in body fluids, they hold promise as potential biomarkers. Several miRNAs are considered to be muscle (myo)-specific, and are thus known as myomiRs. Comparative analyses between dystrophic and normal muscles have identified many interesting changes in myomiR levels. Because the release of myomiRs from dystrophic muscles is likely to occur through secretion and leakage from damaged myofibres, these can be detected in serum or blood, making them interesting blood biomarkers for DMD (Coenen-Stass et al., 2017; Hrach and Mangone, 2019). In addition, exosome-enclosed miRNAs are present in urine. Although in low abundance, this additional promising source of miRNA biomarkers is yet to be thoroughly investigated (Cheng et al., 2014).

Blood and urine biomarkers are of particular interest clinically, as biofluid samples are readily available from patients, unlike the highly invasive muscle biopsy that can be hard to justify. However, we recommend that putative myonecrosis biomarkers in blood or urine be assessed preclinically and thoroughly correlated with histological measurements of myonecrosis in tissues to ensure their validity.

## Inflammation

**Fig. 3.**
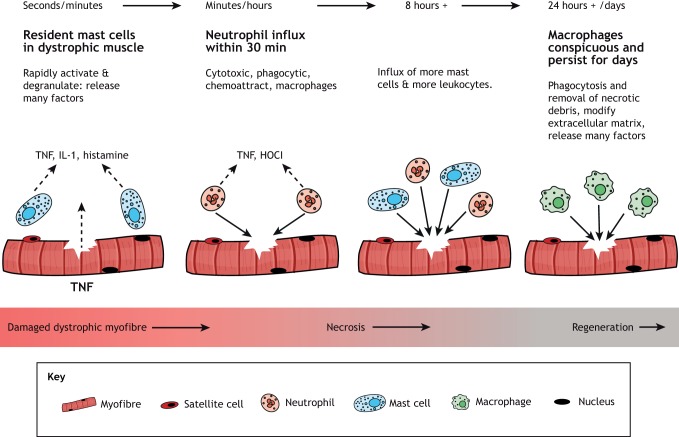
**Sequence of the early inflammatory response to damage in dystrophic skeletal muscles.** Resident mast cells (high in dystrophic muscles) rapidly degranulate to release TNF and many other pro-inflammatory mediators, combined with neutrophils rapidly arriving to produce reactive oxygen species and many other factors, followed by macrophages that persist for several days (adapted from Radley and Grounds, 2006). This image is not published under the terms of the CC-BY license of this article. For permission to reuse, please see Radley and Grounds (2006).

Inflammation is closely associated with myonecrosis in dystrophy, and is therefore discussed because of the potential to link inflammatory biomarkers to myonecrosis. The key cells of the inflammatory response (reviewed by Tidball et al., 2018) that we consider are mast cells, neutrophils (polymorphonuclear leukocytes) and macrophages (Fig. 3). Eosinophils are not considered to be major players in the dystropathology (Sek et al., 2019).

**Fig. 4.**
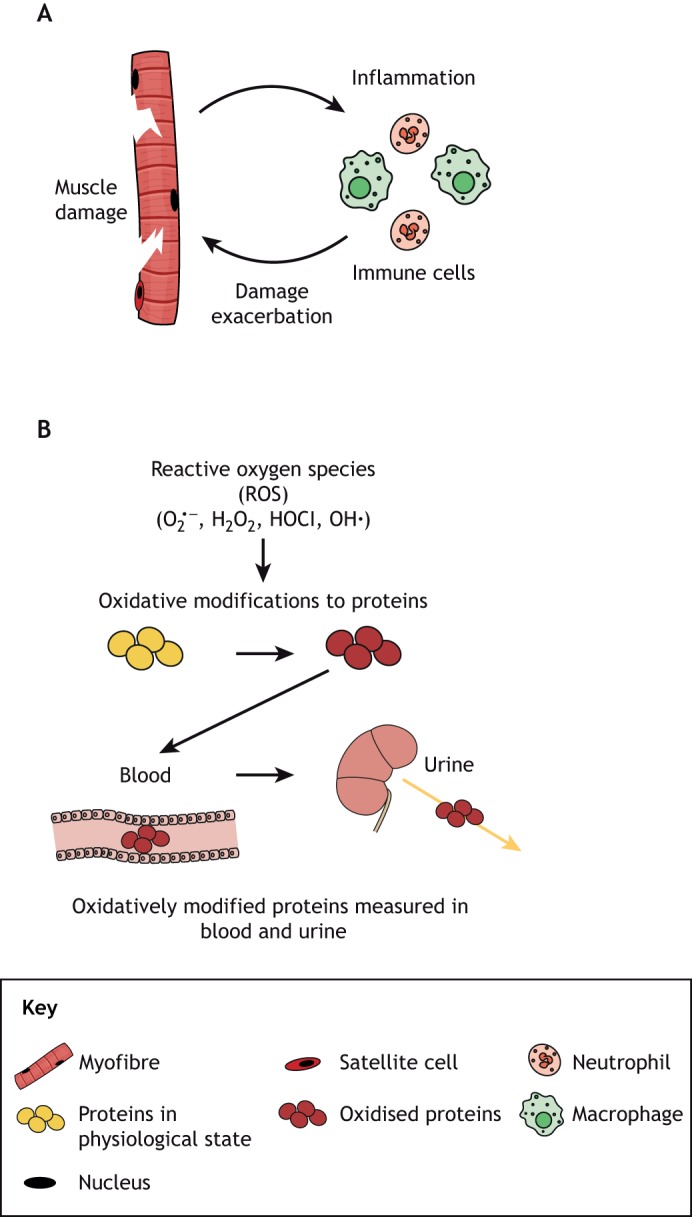
**Generation of reactive oxidative species at the surface of myofibre by neutrophils.** (A) Myofibre damage leads to the infiltration of immune cells to the site of damage, and these cells, particularly neutrophils, have the potential to exacerbate muscle damage by the generation of oxidants. (B) Activation of neutrophils results in the production of superoxide 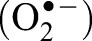, dismutation of which leads to the formation of hydrogen peroxide (H_2_O_2_) that is either catalysed by MPO to form the highly cytotoxic oxidant hypochlorous acid (HOCl), or is further oxidised to generate hydroxyl radicals (OH•). These oxidants can potentially exacerbate necrosis of dystrophic myofibres by the reversible and irreversible damage modifications that affect the function of cellular proteins. These modified proteins can enter circulation and are often excreted, therefore the measurement of these modifications in plasma and urine can be used as biomarkers of inflammation and oxidative stress in the muscle.

In mice, the first cells to exit the vasculature and arrive at the site of experimental damage to normal (non-dystrophic) skeletal muscles are neutrophils, within ∼30 min (Radley and Grounds, 2006; Tidball et al., 2018). They are phagocytic and are the main cells to secrete the enzyme myeloperoxidase (MPO) that oxidises chloride in the presence of hydrogen peroxide (H_2_O_2_) to form the potent antibacterial oxidant hypochlorous acid (HOCl). These oxidants can modify proteins, which are of interest as biomarkers (discussed below and see Fig. 4). Neutrophils are usually transitory in damaged non-dystrophic tissue and produce a range of pro-inflammatory cytokines and chemotactic molecules that attract macrophages to the damage site. These are usually conspicuous from about day 1 for ∼1 week (Grounds and Davies, 1996; Robertson et al., 1993). Dystrophic mouse and dog muscles have elevated neutrophil content, which is likely a consequence of ongoing bouts of myonecrosis ([Bibr DMM043638C104][Bibr DMM043638C104],b,c).

Although there are few mast cells in non-dystrophic mouse muscle, they accumulate in the tissue by ∼8 h after damage and then persist in the damaged tissue. Hence, large numbers of resident mast cells are a feature of dystrophic muscles, with analyses in *mdx* mice reporting ∼9-13 mast cells/mm^2^ muscle (Radley and Grounds, 2006). Mast cells are packed with granules containing many molecules, including histamine and the pro-inflammatory cytokine TNF, that are rapidly released in response to trauma and exacerbate the necrosis of dystrophic myofibres (Tidball et al., 2018).

Macrophages are the main inflammatory cells in damaged non-dystrophic mouse muscle from ∼24 h after experimental injury, peaking at ∼3 days post-damage, which is around the time of intense myoblast proliferation and onset of fusion, and decreasing by 7 days. Different macrophage subtypes play major roles in the early and later stages of muscle regeneration (Tidball et al., 2018). Simplistically, pro-inflammatory macrophages (sometimes referred to as M1) are essential for the critical early events of phagocytosis, remodelling of the ECM, and myogenesis with myotube formation; and macrophages that secrete anti-inflammatory cytokines (M2) are essential for the maturation of the new myofibres and for the resolution of the regenerative process (Tidball et al., 2018). This distinction is complicated in dystrophic muscles, in which overlapping bouts of myonecrosis and regeneration result in disturbed populations of inflammatory cells, with altered effects on various cell types and ‘asynchronous’ regeneration (Dadgar et al., 2014).

Inflammatory cells produce many cytokines, and increased blood levels of pro-inflammatory molecules are potential biomarkers. Increased levels of TNF in dystrophic muscle are of particular interest, as TNF exacerbates myonecrosis and studies show that reducing the levels of TNF using various strategies effectively prevents myonecrosis (see Fig. 2; Hodgetts et al., 2006). Delivering taurine, an amino acid that is abundant in milk, to juvenile pre-weaned *mdx* mice prevented the acute onset of myonecrosis at 22 days and decreased the TNF levels, neutrophil content and MPO activity typically seen in untreated *mdx* muscles upon weaning (Terrill et al., 2016c). Furthermore, co-administration of deflazacort, an anti-inflammatory and immunosuppressant glucocorticoid commonly used to treat DMD patients, and omega-3 fatty acids to *mdx* mice resulted in reduced levels of serum TNF (de Carvalho et al., 2018). Taken together, these studies indicate that changes in blood TNF levels are reflecting the changes in tissue pathology and treatment responsiveness, supporting the notion that blood TNF is a potentially useful clinical biomarker. A potential caveat is that serum TNF levels are relatively low, which traditionally made it difficult to measure using antibodies alone (Saito et al., 2000), but this is now attainable using new advanced technologies (Koelman et al., 2019).

## Oxidative stress

Increased oxidative stress is evident in dystrophic muscle, and is strongly associated with myonecrosis and inflammation (Tidball et al., 2018). Proposed sources of various oxidants include mitochondria, inflammatory cells, NAD(P)H oxidase, xanthine oxidase (Box 1) and decoupling of nitric oxidase synthase (NOS; Box 1) via dislocation or translocation of neuronal (n)NOS from the dystroglycan complex of the sarcolemma (Kim et al., 2013). Our discussion will focus on the oxidative stress associated with inflammation, specifically with neutrophils, as our research group has identified several promising biomarkers associated with these pathways for animal models of DMD.

### Irreversible oxidative damage of macromolecules

One major cellular consequence of oxidant exposure is irreversible damage to proteins and lipids. These are measured by assaying for carbonyls and damaged lipids such as malondialdehyde and isoprostanes (Wilson et al., 2017), which are all elevated in DMD muscle (Haycock et al., 1996; Kar and Pearson, 1979; Mechler et al., 1984; Renjini et al., 2012). Muscles of *mdx* mice show significantly elevated levels of protein carbonyls (but not malondialdehyde) by 24 days of age, as do GRMD dogs by 8 months of age (El-Shafey et al., 2011; Terrill et al., 2016b).

Activated neutrophils generate the potent oxidant HOCl via MPO-mediated peroxidation of chloride ions. As the carbonyl assay is a non-specific measure of oxidant activity, the extent to which HOCl changes carbonyl formation is unknown. A more direct measure of HOCl-mediated oxidative damage is halogenation of protein tyrosine residues (Winterbourn, 2002). When tyrosine-containing peptides and proteins are exposed to HOCl, the resulting chlorotyrosines can be measured by liquid or gas chromatography with mass spectrometry or by immunoblotting using an antibody that can detect halogenated tyrosine (Kato et al., 2005; Winterbourn, 2002). We have shown an increase in tyrosine halogenation in GRMD muscle (Terrill et al., 2016a).

### Reversible oxidation of protein thiols

Thiol oxidation involves the thiol (-SH) groups of cysteine residues on proteins that can undergo numerous reactions, which depend on the specific type and concentration of the oxidants they encounter (Eaton, 2006; Iwasaki et al., 2013; Zuo and Pannell, 2015). Reversible oxidation of thiol groups can affect the function of many proteins that in turn can affect several cellular pathways including proliferation, differentiation, necrosis and contractility (reviewed by Paulsen and Carroll, 2010). Thiol oxidation of proteins has been linked with many diseases, including cardiovascular and pulmonary pathologies (Oliveira and Laurindo, 2018; Zinellu et al., 2016). Although irreversible oxidative damage of proteins and lipids resulting from oxidant exposure has been widely studied and targeted by antioxidant treatment (Halliwell, 2013), there has been relatively little information related to the extent of reversible protein thiol oxidation in muscular dystrophies.

Recent research in animal models of DMD shows striking increases in protein thiol oxidation in dystrophic skeletal muscles (El-Shafey et al., 2011; Iwasaki et al., 2013; Pinniger et al., 2017; Radley-Crabb et al., 2012; Terrill et al., 2012, [Bibr DMM043638C102][Bibr DMM043638C102],b, [Bibr DMM043638C104][Bibr DMM043638C104],b, 2017). We propose that neutrophils are a major source of protein thiol oxidants such as HOCl that exacerbate myonecrosis in dystrophic muscles (as indicated in Fig. 4). Consistent with this, levels of the classic markers for neutrophils, MPO and neutrophil elastase, closely correspond with elevated protein carbonylation, chlorotyrosine formation and thiol oxidation markers in dystrophic skeletal muscles of *mdx* mice and GRMD dogs (Terrill et al., 2016a). We have also shown that reversible protein thiol oxidation is especially localised in foci of myonecrosis (Iwasaki et al., 2013), and occurs on muscle proteins such as myosin heavy chain, myosin light chain and tropomyosin, as well as on the glycolytic proteins phosphoglycerate mutase and triosephosphate isomerase (Armstrong et al., 2011; El-Shafey et al., 2011; Iwasaki et al., 2013; Radley-Crabb et al., 2012; Terrill et al., 2012, [Bibr DMM043638C102][Bibr DMM043638C102],b, [Bibr DMM043638C104][Bibr DMM043638C104]).

**Fig. 5.**
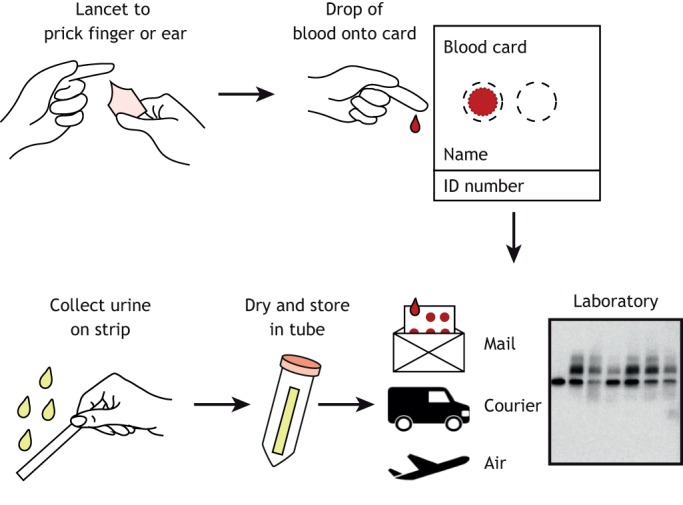
**Home blood and urine collection to measure biomarkers of dystropathology.** Biomarkers that can be measured in a drop of blood collected from a finger prick onto a card for storage at room temperature can be readily collected in the home by patients or their family. Similarly, analytes of dystropathology in urine can potentially be measured using an absorbent strip that is dried and stored at room temperature. Home collection would augment clinical utility by facilitating tracking of biomarkers.

Protein thiol groups are particularly susceptible to oxidation by HOCl, with HOCl estimated to be ∼10^8^× more reactive with thiol groups than H_2_O_2_ (Davies, 2016)_._ Accordingly, proteins containing thiol groups are potential biomarkers of oxidative stress and associated myonecrosis. In plasma, most thiol groups in proteins are in an oxidised state; however, the thiol group of cysteine 34 (Cys34) in human serum albumin is only partially oxidised. As a consequence, albumin Cys34 can be further oxidised, and assays to measure the thiol oxidation state of this amino acid residue have been developed for use as a plasma biomarker of oxidative stress (Colombo et al., 2012; Era et al., 1988; Lamprecht et al., 2008; Lim et al., 2020). It is well documented that various diseases and physiological stresses such as exercise can increase Cys34 oxidation (Nagumo et al., 2014). Therefore, albumin Cys34 has the potential to be a useful plasma biomarker of inflammation and oxidative stress to track myonecrosis. To validate albumin Cys34 as a clinical biomarker for myonecrosis in DMD, our group is investigating the correlation between thiol oxidation of albumin Cys34 in plasma and protein thiol oxidation in the muscle tissue of mouse models. We are also testing an alternate blood collection approach to measure the oxidation state of albumin Cys34, to develop a test that could be more accessible to the wider research and clinical community (Fig. 5).

## Conclusions

As discussed in this Review, many potential molecular biomarkers have been identified in blood or urine of animal models and DMD patients (see Table 1). However, there are many factors to consider when developing biomarkers that are fit for purpose (see Box 2, Factors influencing the development of a clinical biomarker). In particular, and as discussed, there are advantages to linking biomarkers to mechanistically relevant changes in muscle.

**Box 2. Factors influencing the development of a clinical biomarker**

**Collection:** The type of collection for blood (finger prick versus venepuncture, and plasma versus serum) and urine (one-off versus 24 h) affects cost and the ability to collect serial samples to monitor changes in disease severity. Variability during the day, and between days, can affect the time and the number of serial samples required to reliably track changes in the biomarker (Aronson, 2005; FDA, 2016; Florence et al., 1985; Scotton et al., 2014).

**Stability:** Biomarkers stable at room temperature would not require the expense and logistical challenges of maintaining and transporting cold or frozen samples (Kraus et al., 2015; LaBaer, 2005).

**Analytical:** Techniques for analysis that do not require specialised equipment and staff would enhance clinical utility by decreasing costs and facilitate dissemination of the test to different laboratories. Reliable performance metrics (e.g. accuracy, precision and reproducibility), particularly across several laboratories, would also support regulatory approval as a drug development tool (Kraus, 2018).

**Validation:** Linking biofluid biomarker changes to measures of myonecrosis, particularly with preclinical treatments, would provide evidence for clinical validity (Kraus, 2018).

**Clinical utility:** Clinical utility describes how well a test balances likely benefit and potential disadvantages when used in patient management and for use in drug development trials (Kraus, 2018).

Although human blood and urine samples can be fairly easy to obtain for analyses, tracking changes in muscles is difficult because muscle biopsies are highly invasive and undesirable for DMD boys. However, animal models provide the opportunity to compare changes in the levels of a biomarker relative to the level of myonecrosis from the same individual. Given the limitations of the dystrophic animal models, including duration of growth and lifespan, relative size and loading of muscles, it is desirable to test and validate promising biomarkers across several dystrophic species. After validation in muscles across dystrophic animal species (Box 2) and their identification and validation in blood, plasma and urine of the same animal models, robust biomarkers can be tested clinically in DMD and age-matched normal control biofluids. Such data strengthen the case for a biomarker as a meaningful clinical readout, and this approach could be more widely applied to help validate robust biomarkers for DMD.

Drug development and clinical trials have become increasingly complex and resource-intensive, with strong competition for access to relatively small numbers of vulnerable young DMD patients. These challenges could in part be alleviated by using myonecrosis-tracking and other relevant biomarkers in biofluids to critically and relatively rapidly assess the benefits of candidate therapies, and help to prioritise and accelerate the most promising clinical therapies for DMD and other neuromuscular diseases.

This article is part of a special collection ‘A Guide to Using Neuromuscular Disease Models for Basic and Preclinical Studies’, which was launched in a dedicated issue guest edited by Annemieke Aartsma-Rus, Maaike van Putten and James Dowling. See related articles in this collection at http://dmm.biologists.org/collection/neuromuscular.

## References

[DMM043638C1] Aartsma-Rus A. and Spitali P. (2015). Circulating biomarkers for Duchenne muscular dystrophy. *J. Neuromuscul. Dis.* 2, S49-S58. 10.3233/JND-150102 PMC5271432 27858763

[DMM043638C2] Aartsma-Rus A., Ferlini A., McNally E. M., Spitali P., Sweeney H. L. and Workshop participants (2018). 226(th) ENMC International Workshop: Towards validated and qualified biomarkers for therapy development for Duchenne muscular dystrophy 20-22 January 2017, Heemskerk, The Netherlands. *Neuromuscul. Disord.* 28, 77-86. 10.1016/j.nmd.2017.10.002 29203356 PMC5957286

[DMM043638C3] Allen D. G., Whitehead N. P. and Froehner S. C. (2016). Absence of Dystrophin disrupts skeletal muscle signaling: roles of Ca^2^+, reactive oxygen species, and nitric oxide in the development of muscular dystrophy. *Physiol. Rev.* 96, 253-305. 10.1152/physrev.00007.2015 26676145 PMC4698395

[DMM043638C4] Amthor H., Egelhof T., McKinnell I., Ladd M. E., Janssen I., Weber J., Sinn H., Schrenk H.-H., Forsting M., Voit T. et al. (2004). Albumin targeting of damaged muscle fibres in the mdx mouse can be monitored by MRI. *Neuromuscul. Disord.* 14, 791-796. 10.1016/j.nmd.2004.08.004 15564034

[DMM043638C5] Armstrong A. E., Zerbes R., Fournier P. A. and Arthur P. G. (2011). A fluorescent dual labeling technique for the quantitative measurement of reduced and oxidized protein thiols in tissue samples. *Free Radic. Biol. Med.* 50, 510-517. 10.1016/j.freeradbiomed.2010.11.018 21109000

[DMM043638C6] Aronson J. K. (2005). Biomarkers and surrogate endpoints. *Br. J. Clin. Pharmacol.* 59, 491-494. 10.1111/j.1365-2125.2005.02435.x 15842546 PMC1884846

[DMM043638C7] Arthur P. G., Grounds M. D. and Shavlakadze T. (2008). Oxidative stress as a therapeutic target during muscle wasting: considering the complex interactions. *Curr. Opin. Clin. Nutr. Metab. Care* 11, 408-416. 10.1097/MCO.0b013e328302f3fe 18542000

[DMM043638C8] Barker C. I. S., Standing J. F., Kelly L. E., Hanly Faught L., Needham A. C., Rieder M. J., de Wildt S. N. and Offringa M. (2018). Pharmacokinetic studies in children: recommendations for practice and research. *Arch. Dis. Child.* 103, 695-702. 10.1136/archdischild-2017-314506 29674514 PMC6047150

[DMM043638C9] Biggar W. D. (2006). Duchenne muscular dystrophy. *Pediatr. Rev.* 27, 83-88. 10.1542/pir.27-3-83 16510548

[DMM043638C10] Blaveri K., Heslop L., Yu D. S., Rosenblatt J. D., Gross J. G., Partridge T. A. and Morgan J. E. (1999). Patterns of repair of dystrophic mouse muscle: studies on isolated fibers. *Dev. Dyn.* 216, 244-256. 10.1002/(SICI)1097-0177(199911)216:3<244::AID-DVDY3>3.0.CO;2-9 10590476

[DMM043638C11] Bushby K., Finkel R., Birnkrant D. J., Case L. E., Clemens P. R., Cripe L., Kaul A., Kinnett K., McDonald C., Pandya S. et al. (2010). Diagnosis and management of Duchenne muscular dystrophy, part 2: implementation of multidisciplinary care. *Lancet Neurol.* 9, 177-189. 10.1016/S1474-4422(09)70272-8 19945914

[DMM043638C12] Butchart L. C., Terrill J. R., Rossetti G., White R., Filipovska A. and Grounds M. D. (2018). Expression patterns of regulatory RNAs, including lncRNAs and tRNAs, during postnatal growth of normal and dystrophic (mdx) mouse muscles, and their response to Taurine treatment. *Int. J. Biochem. Cell Biol.* 99, 52-63. 10.1016/j.biocel.2018.03.016 29578051

[DMM043638C13] Chen Y.-W., Nagaraju K., Bakay M., McIntyre O., Rawat R., Shi R. and Hoffman E. P. (2005). Early onset of inflammation and later involvement of TGFβ in Duchenne muscular dystrophy. *Neurology* 65, 826-834. 10.1212/01.wnl.0000173836.09176.c4 16093456

[DMM043638C14] Cheng L., Sun X., Scicluna. J., Coleman B. M. and Hill A. F. (2014). Characterization and deep sequencing analysis of exosomal and non-exosomal miRNA in human urine. *Kidney Int.* 86, 433-444. 10.1038/ki.2013.502 24352158

[DMM043638C15] Coenen-Stass A. M. L., Wood M. J. A. and Roberts T. C. (2017). Biomarker Potential of Extracellular miRNAs in Duchenne muscular dystrophy. *Trends Mol. Med.* 23, 989-1001. 10.1016/j.molmed.2017.09.002 28988850

[DMM043638C16] Colombo G., Clerici M., Giustarini D., Rossi R., Milzani A. and Dalle-Donne I. (2012). Redox albuminomics: oxidized albumin in human diseases. *Antioxid. Redox Signal.* 17, 1515-1527. 10.1089/ars.2012.4702 22587567

[DMM043638C17] Coulton G. R., Morgan J. E., Partridge T. A. and Sloper J. C. (1988). The mdx mouse skeletal muscle myopathy: I. A histological, morphometric and biochemical investigation. *Neuropathol. Appl. Neurobiol.* 14, 53-70. 10.1111/j.1365-2990.1988.tb00866.x 2967442

[DMM043638C18] Cruz-Guzman Odel R., Rodriguez-Cruz M. and Escobar Cedillo R. E. (2015). Systemic inflammation in Duchenne muscular dystrophy: association with muscle function and nutritional status. *Biomed. Res. Int.* 2015, 891972 10.1155/2015/891972 26380303 PMC4561314

[DMM043638C19] Cullen M. J. and Fulthorpe J. J. (1975). Stages in fibre breakdown in Duchenne muscular dystrophy. An electron-microscopic study. *J. Neurol. Sci.* 24, 179-200. 10.1016/0022-510X(75)90232-4 163299

[DMM043638C20] Cynthia Martin F., Hiller M., Spitali P., Oonk S., Dalebout H., Palmblad M., Chaouch A., Guglieri M., Straub V., Lochmüller H. et al. (2014). Fibronectin is a serum biomarker for Duchenne muscular dystrophy. *Proteomics Clin. Appl.* 8, 269-278. 10.1002/prca.201300072 24458521

[DMM043638C21] Dadgar S., Wang Z., Johnston H., Kesari A., Nagaraju K., Chen Y.-W., Hill D. A., Partridge T. A., Giri M., Freishtat R. J. et al. (2014). Asynchronous remodeling is a driver of failed regeneration in Duchenne muscular dystrophy. *J. Cell Biol.* 207, 139-158. 10.1083/jcb.201402079 25313409 PMC4195829

[DMM043638C22] Davies M. J. (2016). Protein oxidation and peroxidation. *Biochem. J.* 473, 805-825. 10.1042/BJ20151227 27026395 PMC4819570

[DMM043638C23] de Carvalho S. C., Matsumura C. Y., Santo Neto H. and Marques M. J. (2018). Identification of plasma interleukins as biomarkers for deflazacort and omega-3 based Duchenne muscular dystrophy therapy. *Cytokine* 102, 55-61. 10.1016/j.cyto.2017.12.006 29276972

[DMM043638C24] Dowling P., Murphy S., Zweyer M., Raucamp M., Swandulla D. and Ohlendieck K. (2019). Emerging proteomic biomarkers of X-linked muscular dystrophy. *Expert Rev. Mol. Diagn.* 19, 739-755. 10.1080/14737159.2019.1648214 31359811

[DMM043638C25] Eaton P. (2006). Protein thiol oxidation in health and disease: techniques for measuring disulfides and related modifications in complex protein mixtures. *Free Radic. Biol. Med.* 40, 1889-1899. 10.1016/j.freeradbiomed.2005.12.037 16716890

[DMM043638C26] El-Shafey A. F., Armstrong A. E., Terrill J. R., Grounds M. D. and Arthur P. G. (2011). Screening for increased protein thiol oxidation in oxidatively stressed muscle tissue. *Free Radic. Res.* 45, 991-999. 10.3109/10715762.2011.590136 21696323

[DMM043638C27] Emery A. E. H. (2002). The muscular dystrophies. *Lancet* 359, 687-695. 10.1016/S0140-6736(02)07815-7 11879882

[DMM043638C28] Era S., Hamaguchi T., Sogami M., Kuwata K., Suzuki E., Miura K., Kawai K., Kitazawa Y., Okabe H., Noma A. et al. (1988). Further studies on the resolution of human mercapt- and nonmercaptalbumin and on human serum albumin in the elderly by high-performance liquid chromatography. *Int. J. Pept. Protein Res.* 31, 435-442. 10.1111/j.1399-3011.1988.tb00900.x 3410634

[DMM043638C29] Falzarano M. S., Scotton C., Passarelli C. and Ferlini A. (2015). Duchenne muscular dystrophy: from diagnosis to therapy. *Molecules* 20, 18168-18184. 10.3390/molecules201018168 26457695 PMC6332113

[DMM043638C30] Fanin M., Nascimbeni A. C. and Angelini C. (2014). Muscle atrophy, ubiquitin-proteasome, and autophagic pathways in dysferlinopathy. *Muscle Nerve* 50, 340-347. 10.1002/mus.24167 24395438

[DMM043638C31] FDA. (2016). *Considerations for Use of Histopathology and its Associated Methodologies to Support Biomarker Qualification*. US Food and Drug Administration.

[DMM043638C32] Fitzsimons R. B. and Hoh J. F. (1981). Embryonic and foetal myosins in human skeletal muscle. The presence of foetal myosins in duchenne muscular dystrophy and infantile spinal muscular atrophy. *J. Neurol. Sci.* 52, 367-384. 10.1016/0022-510X(81)90018-6 7310440

[DMM043638C33] Florence J. M., Fox P. T., Planer G. J. and Brooke M. H. (1985). Activity. creatine kinase, and myoglobin in Duchenne muscular dystrophy: a clue to etiology? *Neurology* 35, 758-761. 10.1212/WNL.35.5.758 4039424

[DMM043638C35] Gordish-Dressman H., Willmann R., Dalle Pazze L., Kreibich A., van Putten M., Heydemann A., Bogdanik L., Lutz C., Davies K., Demonbreun A. R. et al. (2018). “Of mice and measures”: a project to improve how we advance Duchenne muscular dystrophy therapies to the clinic. *J. Neuromuscul. Dis.* 5, 407-417. 10.3233/JND-180324 30198876 PMC6218134

[DMM043638C36] Greco S., De Simone M., Colussi C., Zaccagnini G., Fasanaro P., Pescatori M., Cardani R., Perbellini R., Isaia E., Sale P. et al. (2009). Common micro-RNA signature in skeletal muscle damage and regeneration induced by Duchenne muscular dystrophy and acute ischemia. *FASEB J.* 23, 3335-3346. 10.1096/fj.08-128579 19528256

[DMM043638C37] Grounds M. D. (2008). Two-tiered hypotheses for Duchenne muscular dystrophy. *Cell Mol. Life Sci.* 65, 1621-1625. 10.1007/s00018-008-7574-8 18327663 PMC11131677

[DMM043638C38] Grounds M. D. (2014). The need to more precisely define aspects of skeletal muscle regeneration. *Int. J. Biochem. Cell Biol.* 56, 56-65. 10.1016/j.biocel.2014.09.010 25242742

[DMM043638C39] Grounds M. D. and Davies M. J. (1996). Chemotaxis in myogenesis. *Mol. Biol. Cell* 7, 3758-3758.

[DMM043638C40] Haddix S. G., Lee Y. I., Kornegay J. N. and Thompson W. J. (2018). Cycles of myofiber degeneration and regeneration lead to remodeling of the neuromuscular junction in two mammalian models of Duchenne muscular dystrophy. *PLoS ONE* 13, e0205926 10.1371/journal.pone.0205926 30379896 PMC6209224

[DMM043638C41] Halliwell B. (2013). The antioxidant paradox: less paradoxical now? *Br. J. Clin. Pharmacol.* 75, 637-644. 10.1111/j.1365-2125.2012.04272.x 22420826 PMC3575931

[DMM043638C42] Hamer P. W., McGeachie J. M., Davies M. J. and Grounds M. D. (2002). Evans Blue Dye as an in vivo marker of myofibre damage: optimising parameters for detecting initial myofibre membrane permeability. *J. Anat.* 200, 69-79. 10.1046/j.0021-8782.2001.00008.x 11837252 PMC1570883

[DMM043638C43] Hathout Y., Marathi R. L., Rayavarapu S., Zhang A., Brown K. J., Seol H., Gordish-Dressman H., Cirak S., Bello L., Nagaraju K. et al. (2014). Discovery of serum protein biomarkers in the mdx mouse model and cross-species comparison to Duchenne muscular dystrophy patients. *Hum. Mol. Genet.* 23, 6458-6469. 10.1093/hmg/ddu366 25027324 PMC4240201

[DMM043638C44] Hathout Y., Seol H., Han M. H. J., Zhang A., Brown K. J. and Hoffman E. P. (2016). Clinical utility of serum biomarkers in Duchenne muscular dystrophy. *Clin. Proteomics* 13, 9 10.1186/s12014-016-9109-x 27051355 PMC4820909

[DMM043638C45] Haycock J. W., MacNeil S., Jones P., Harris J. B. and Mantle D. (1996). Oxidative damage to muscle protein in Duchenne muscular dystrophy. *Neuroreport* 8, 357-361. 10.1097/00001756-199612200-00070 9051810

[DMM043638C46] Hodgetts S., Radley H., Davies M. and Grounds M. D. (2006). Reduced necrosis of dystrophic muscle by depletion of host neutrophils, or blocking TNFalpha function with Etanercept in mdx mice. *Neuromuscul. Disord.* 16, 591-602. 10.1016/j.nmd.2006.06.011 16935507

[DMM043638C47] Hrach H. C. and Mangone M. (2019). miRNA Profiling for early detection and treatment of Duchenne muscular dystrophy. *Int. J. Mol. Sci.* 20, e4638 10.3390/ijms20184638 31546754 PMC6769970

[DMM043638C48] Iwasaki T., Terrill J., Shavlakadze T., Grounds M. D. and Arthur P. G. (2013). Visualizing and quantifying oxidized protein thiols in tissue sections: a comparison of dystrophic mdx and normal skeletal mouse muscles. *Free Radic. Biol. Med.* 65, 1408-1416. 10.1016/j.freeradbiomed.2013.09.024 24095851

[DMM043638C49] John H. A. and Purdom I. F. (1989). Elevated plasma levels of haptoglobin in Duchenne muscular dystrophy:electrophoretic variants in patients with a severe form of the disease. *Electrophoresis* 10, 489-493. 10.1002/elps.1150100707 2776732

[DMM043638C50] Kar N. C. and Pearson C. M. (1979). Catalase, superoxide dismutase, glutathione reductase and thiobarbituric acid-reactive products in normal and dystrophic human muscle. *Clin. Chim. Acta* 94, 277-280. 10.1016/0009-8981(79)90076-7 466816

[DMM043638C51] Kato Y., Kawai Y., Morinaga H., Kondo H., Dozaki N., Kitamoto N. and Osawa T. (2005). Immunogenicity of a brominated protein and successive establishment of a monoclonal antibody to dihalogenated tyrosine. *Free Radic. Biol. Med.* 38, 24-31. 10.1016/j.freeradbiomed.2004.09.013 15589368

[DMM043638C52] Kharraz Y., Guerra J., Pessina P., Serrano A. L. and Muñoz-Cánoves P. (2014). Understanding the process of fibrosis in Duchenne muscular dystrophy. *Biomed. Res. Int.* 2014, 965631 10.1155/2014/965631 24877152 PMC4024417

[DMM043638C53] Kim J.-H., Kwak H.-B., Thompson L. D. V. and Lawler J. M. (2013). Contribution of oxidative stress to pathology in diaphragm and limb muscles with Duchenne muscular dystrophy. *J. Muscle Res. Cell Motil.* 34, 1-13. 10.1007/s10974-012-9330-9 23104273

[DMM043638C54] Koelman L., Pivovarova-Ramich O., Pfeiffer A. F. H., Grune T. and Aleksandrova K. (2019). Cytokines for evaluation of chronic inflammatory status in ageing research: reliability and phenotypic characterisation. *Immun. Ageing* 16, 11 10.1186/s12979-019-0151-1 31139232 PMC6530020

[DMM043638C55] Kornegay J. N. (2017). The golden retriever model of Duchenne muscular dystrophy. *Skelet. Muscle* 7, 9 10.1186/s13395-017-0124-z 28526070 PMC5438519

[DMM043638C56] Kraus V. B. (2018). Biomarkers as drug development tools: discovery, validation, qualification and use. *Nat. Rev. Rheumatol.* 14, 354-362. 10.1038/s41584-018-0005-9 29760435

[DMM043638C57] Kraus V. B., Blanco F. J., Englund M., Henrotin Y., Lohmander L. S., Losina E., Önnerfjord P. and Persiani S. (2015). OARSI clinical trials recommendations: soluble biomarker assessments in clinical trials in osteoarthritis. *Osteoarthritis Cartilage* 23, 686-697. 10.1016/j.joca.2015.03.002 25952342 PMC4430113

[DMM043638C58] Krishnan V. S., White Z., McMahon C. D., Hodgetts S. I., Fitzgerald M., Shavlakadze T., Harvey A. R. and Grounds M. D. (2016). A neurogenic perspective of Sarcopenia: time course study of sciatic nerves from aging mice. *J. Neuropathol. Exp. Neurol.* 75, 464-478. 10.1093/jnen/nlw019 27030741

[DMM043638C59] Kuraoka M., Kimura E., Nagata T., Okada T., Aoki Y., Tachimori H., Yonemoto N., Imamura M. and Takeda S. (2016). Serum osteopontin as a novel biomarker for muscle regeneration in Duchenne muscular dystrophy. *Am. J. Pathol.* 186, 1302-1312. 10.1016/j.ajpath.2016.01.002 26963343

[DMM043638C60] LaBaer J. (2005). So, you want to look for biomarkers (introduction to the special biomarkers issue). *J. Proteome Res.* 4, 1053-1059. 10.1021/pr0501259 16083254

[DMM043638C61] Lamprecht M., Greilberger J. F., Schwaberger G., Hofmann P. and Oettl K. (2008). Single bouts of exercise affect albumin redox state and carbonyl groups on plasma protein of trained men in a workload-dependent manner. *J. Appl. Physiol.* 104, 1611-1617. 10.1152/japplphysiol.01325.2007 18420715

[DMM043638C62] Larcher T., Lafoux A., Tesson L., Remy S., Thepenier V., François V., Le Guiner C., Goubin H., Dutilleul M., Guigand L. et al. (2014). Characterization of dystrophin deficient rats: a new model for Duchenne muscular dystrophy. *PLoS ONE* 9, e110371 10.1371/journal.pone.0110371 25310701 PMC4195719

[DMM043638C63] Li X., Li Y., Zhao L., Zhang D., Yao X., Zhang H., Wang Y.-C., Wang X.-Y., Xia H., Yan J. et al. (2014). Circulating muscle-specific miRNAs in Duchenne muscular dystrophy patients. *Mol. Ther. Nucleic Acids* 3, e177 10.1038/mtna.2014.29 25050825 PMC4121518

[DMM043638C64] Lim Z. X., Duong M. N., Boyatzis A. E., Golden E., Vrielink A., Fournier P. A. and Arthur P. G. (2020). Oxidation of cysteine 34 of plasma albumin as a biomarker of oxidative stress. *Free Radic. Res.* 54, 1-13. 10.1080/10715762.2019.1708347 31903812

[DMM043638C65] Lourbakos A., Yau N., de Bruijn P., Hiller M., Kozaczynska K., Jean-Baptiste R., Reza M., Wolterbeek R., Koeks Z., Ayoglu B. et al. (2017). Evaluation of serum MMP-9 as predictive biomarker for antisense therapy in Duchenne. *Sci. Rep.* 7, 17888 10.1038/s41598-017-17982-y 29263366 PMC5738430

[DMM043638C66] Mechler F., Imre S. and Dioszeghy P. (1984). Lipid peroxidation and superoxide dismutase activity in muscle and erythrocytes in Duchenne muscular dystrophy. *J. Neurol. Sci.* 63, 279-283. 10.1016/0022-510X(84)90150-3 6726273

[DMM043638C67] Miike T., Sugino S., Ohtani Y., Taku K. and Yoshioka K. (1987). Vascular endothelial cell injury and platelet embolism in Duchenne muscular dystrophy at the preclinical stage. *J. Neurol. Sci.* 82, 67-80. 10.1016/0022-510X(87)90007-4 3440873

[DMM043638C68] Misaka T., Yoshihisa A. and Takeishi Y. (2019). Titin in muscular dystrophy and cardiomyopathy: urinary titin as a novel marker. *Clin. Chim. Acta* 495, 123-128. 10.1016/j.cca.2019.04.005 30959043

[DMM043638C69] Moat S. J., Korpimäki T., Furu P., Hakala H., Polari H., Meriö L., Mäkinen P. and Weeks I. (2017). Characterization of a blood spot creatine kinase skeletal muscle isoform immunoassay for high-throughput newborn screening of Duchenne muscular dystrophy. *Clin. Chem.* 63, 908-914. 10.1373/clinchem.2016.268425 28209627

[DMM043638C70] Morgan J. E., Prola A., Mariot V., Pini V., Meng J., Hourde C., Dumonceaux J., Conti F., Relaix F., Authier F.-J. et al. (2018). Necroptosis mediates myofibre death in dystrophin-deficient mice. *Nat. Commun.* 9, 3655 10.1038/s41467-018-06057-9 30194302 PMC6128848

[DMM043638C71] Nadarajah V. D., van Putten M., Chaouch A., Garrood P., Straub V., Lochmüller H., Ginjaar H. B., Aartsma-Rus A. M., van Ommen G. J. B., den Dunnen J. T. and et al. (2011). Serum matrix metalloproteinase-9 (MMP-9) as a biomarker for monitoring disease progression in Duchenne muscular dystrophy (DMD). *Neuromuscul. Disord.* 21, 569-578. 10.1016/j.nmd.2011.05.011 21724396

[DMM043638C72] Nagumo K., Tanaka M., Chuang V. T. G., Setoyama H., Watanabe H., Yamada N., Kubota K., Tanaka M., Matsushita K., Yoshida A. et al. (2014). Cys34-cysteinylated human serum albumin is a sensitive plasma marker in oxidative stress-related chronic diseases. *PLoS ONE* 9, e85216 10.1371/journal.pone.0085216 24416365 PMC3885702

[DMM043638C73] Oliveira P. V. S. and Laurindo F. R. M. (2018). Implications of plasma thiol redox in disease. *Clin. Sci.* 132, 1257-1280. 10.1042/CS20180157 29967247

[DMM043638C74] Palladino M., Gatto I., Neri V., Straino S., Smith R. C., Silver M., Gaetani E., Marcantoni M., Giarretta I., Stigliano E. et al. (2013). Angiogenic impairment of the vascular endothelium: a novel mechanism and potential therapeutic target in muscular dystrophy. *Arterioscler. Thromb. Vasc. Biol.* 33, 2867-2876. 10.1161/ATVBAHA.112.301172 24072696

[DMM043638C75] Parolo S., Marchetti L., Lauria M., Misselbeck K., Scott-Boyer M.-P., Caberlotto L. and Priami C. (2018). Combined use of protein biomarkers and network analysis unveils deregulated regulatory circuits in Duchenne muscular dystrophy. *PLoS ONE* 13, e0194225 10.1371/journal.pone.0194225 29529088 PMC5846794

[DMM043638C76] Partridge T. A. (2011). Impending therapies for Duchenne muscular dystrophy. *Curr. Opin. Neurol.* 24, 415-422. 10.1097/WCO.0b013e32834aa3f1 21892079

[DMM043638C77] Partridge T. A. (2013). The mdx mouse model as a surrogate for Duchenne muscular dystrophy. *FEBS J.* 280, 4177-4186. 10.1111/febs.12267 23551987 PMC4147949

[DMM043638C78] Paulsen C. E. and Carroll K. S. (2010). Orchestrating redox signaling networks through regulatory cysteine switches. *Acs Chem. Biol.* 5, 47-62. 10.1021/cb900258z 19957967 PMC4537063

[DMM043638C80] Percy M. E., Andrews D. F. and Thompson M. W. (1982). Serum creatine kinase in the detection of Duchenne muscular dystrophy carriers: effects of season and multiple testing. *Muscle Nerve* 5, 58-64. 10.1002/mus.880050111 7057807

[DMM043638C81] Pinniger G. J., Terrill J. R., Assan E. B., Grounds M. D. and Arthur P. G. (2017). Pre-clinical evaluation of N-acetylcysteine reveals side effects in the mdx mouse model of Duchenne muscular dystrophy. *J. Physiol.* 595, 7093-7107. 10.1113/JP274229 28887840 PMC5709333

[DMM043638C82] Radley H. G. and Grounds M. D. (2006). Cromolyn administration (to block mast cell degranulation) reduces necrosis of dystrophic muscle in mdx mice. *Neurobiol. Dis.* 23, 387-397. 10.1016/j.nbd.2006.03.016 16798005

[DMM043638C83] Radley-Crabb H. G., Fiorotto M. L. and Grounds M. D. (2011). The different impact of a high fat diet on dystrophic mdx and control C57Bl/10 mice. *PLoS Curr.* 3, RRN1276 10.1371/currents.RRN1276 22094293 PMC3217191

[DMM043638C84] Radley-Crabb H. G., Terrill J., Shavlakadze T., Tonkin J., Arthur P. and Grounds M. (2012). A single 30 min treadmill exercise session is suitable for ‘proof-of concept studies’ in adult mdx mice: a comparison of the early consequences of two different treadmill protocols. *Neuromuscul. Disord.* 22, 170-182. 10.1016/j.nmd.2011.07.008 21835619

[DMM043638C85] Radley-Crabb H. G., Marini J. C., Sosa H. A., Castillo L. I., Grounds M. D. and Fiorotto M. L. (2014). Dystropathology increases energy expenditure and protein turnover in the mdx mouse model of duchenne muscular dystrophy. *PLoS ONE* 9, e89277 10.1371/journal.pone.0089277 24586653 PMC3929705

[DMM043638C86] Reagan-Shaw S., Nihal M. and Ahmad N. (2008). Dose translation from animal to human studies revisited. *FASEB J.* 22, 659-661. 10.1096/fj.07-9574LSF 17942826

[DMM043638C87] Renjini R., Gayathri N., Nalini A. and Srinivas Bharath M. M. (2012). Oxidative damage in muscular dystrophy correlates with the severity of the pathology: role of glutathione metabolism. *Neurochem. Res.* 37, 885-898. 10.1007/s11064-011-0683-z 22219131

[DMM043638C88] Ridge J. C., Tidball J. G., Ahl K., Law D. J. and Rickoll W. L. (1994). Modifications in myotendinous junction surface morphology in dystrophin-deficient mouse muscle. *Exp. Mol. Pathol.* 61, 58-68. 10.1006/exmp.1994.1025 7995379

[DMM043638C89] Robertson T. A., Maley M. A. L., Grounds M. D. and Papadimitriou J. M. (1993). The role of macrophages in skeletal muscle regeneration with particular reference to chemotaxis. *Exp. Cell Res.* 207, 321-331. 10.1006/excr.1993.1199 8344384

[DMM043638C90] Rouillon J., Lefebvre T., Denard J., Puy V., Daher R., Ausseil J., Zocevic A., Fogel P., Peoc'h K., Wong B. et al. (2018). High urinary ferritin reflects myoglobin iron evacuation in DMD patients. *Neuromuscul. Disord.* 28, 564-571. 10.1016/j.nmd.2018.03.008 29776718

[DMM043638C91] Saito K., Kobayashi D., Komatsu M., Yajima T., Yagihashi A., Ishikawa Y., Minami R. and Watanabe N. (2000). A sensitive assay of tumor necrosis factor alpha in sera from Duchenne muscular dystrophy patients. *Clin. Chem.* 46, 1703-1704. 10.1093/clinchem/46.10.1703 11017956

[DMM043638C92] Schmalbruch H. (1975). Segmental fibre breakdown and defects of the plasmalemma in diseased human muscles. *Acta Neuropathol.* 33, 129-141. 10.1007/BF00687539 1202896

[DMM043638C93] Scotton C., Passarelli C., Neri M. and Ferlini A. (2014). Biomarkers in rare neuromuscular diseases. *Exp. Cell Res.* 325, 44-49. 10.1016/j.yexcr.2013.12.020 24389168

[DMM043638C94] Sek A. C., Moore I. N., Smelkinson M. G., Pak K., Minai M., Smith R., Ma M., Percopo C. M. and Rosenberg H. F. (2019). Eosinophils Do Not Drive Acute Muscle Pathology in the mdx Mouse Model of Duchenne Muscular Dystrophy. *J. Immunol.* 203, 476-484. 10.4049/jimmunol.1900307 31142604 PMC6615969

[DMM043638C95] Smith L. R., Hammers D. W., Sweeney H. L. and Barton E. R. (2016). Increased collagen cross-linking is a signature of dystrophin-deficient muscle. *Muscle Nerve* 54, 71-78. 10.1002/mus.24998 26616495 PMC5067682

[DMM043638C96] Soltan H. C. and Blanchaer M. C. (1959). Activity of serum aldolase and lactic dehydrogenase in patients affected with Duchenne muscular dystrophy and in their immediate relatives. *J. Pediatr.* 54, 27-33. 10.1016/S0022-3476(59)80033-0 13611611

[DMM043638C97] Spitali P., Hettne K., Tsonaka R., Charrout M., van den Bergen J., Koeks Z., Kan H. E., Hooijmans M. T., Roos A., Straub V. et al. (2018). Tracking disease progression non-invasively in Duchenne and Becker muscular dystrophies. *J. Cachexia Sarcopenia Muscle* 9, 715-726. 10.1002/jcsm.12304 29682908 PMC6104105

[DMM043638C98] Straub V., Rafael J. A., Chamberlain J. S. and Campbell K. P. (1997). Animal models for muscular dystrophy show different patterns of sarcolemmal disruption. *J. Cell Biol.* 139, 375-385. 10.1083/jcb.139.2.375 9334342 PMC2139791

[DMM043638C99] Sui T., Lau Y. S., Liu D., Liu T., Xu L., Gao Y., Lai L., Li Z. and Han R. (2018). A novel rabbit model of Duchenne muscular dystrophy generated by CRISPR/Cas9. *Dis. Model Mech.* 11, dmm032201 10.1242/dmm.032201 29871865 PMC6031364

[DMM043638C100] Szigyarto C. A. and Spitali P. (2018). Biomarkers of Duchenne muscular dystrophy: current findings. *Degener. Neurol. Neuromuscul. Dis.* 8, 1-13. 10.2147/DNND.S121099 30050384 PMC6053903

[DMM043638C101] Terrill J. R., Radley-Crabb H. G., Grounds M. D. and Arthur P. G. (2012). N-Acetylcysteine treatment of dystrophic mdx mice results in protein thiol modifications and inhibition of exercise induced myofibre necrosis. *Neuromuscul. Disord.* 22, 427-434. 10.1016/j.nmd.2011.11.007 22206641

[DMM043638C102] Terrill J. R., Boyatzis A., Grounds M. D. and Arthur P. G. (2013a). Treatment with the cysteine precursor l-2-oxothiazolidine-4-carboxylate (OTC) implicates taurine deficiency in severity of dystropathology in mdx mice. *Int. J. Biochem. Cell Biol.* 45, 2097-2108. 10.1016/j.biocel.2013.07.009 23892094

[DMM043638C103] Terrill J. R., Radley-Crabb H. G., Iwasaki T., Lemckert F. A., Arthur P. G. and Grounds M. D. (2013b). Oxidative stress and pathology in muscular dystrophies: focus on protein thiol oxidation and dysferlinopathies. *FEBS J.* 280, 4149-4164. 10.1111/febs.12142 23332128

[DMM043638C104] Terrill J. R., Duong M. N., Turner R., Le Guiner C., Boyatzis A., Kettle A. J., Grounds M. D. and Arthur P. G. (2016a). Levels of inflammation and oxidative stress, and a role for taurine in dystropathology of the golden retriever muscular dystrophy dog model for Duchenne muscular dystrophy. *Redox Biol.* 9, 276-286. 10.1016/j.redox.2016.08.016 27611888 PMC5018082

[DMM043638C105] Terrill J. R., Pinniger G. J., Graves J. A., Grounds M. D. and Arthur P. G. (2016b). Increasing taurine intake and taurine synthesis improves skeletal muscle function in the mdx mouse model for Duchenne muscular dystrophy. *J. Physiol.* 594, 3095-3110. 10.1113/JP271418 26659826 PMC4887673

[DMM043638C106] Terrill J. R. P., Grounds M. D. and Arthur P. G. (2016c). Increased taurine in pre-weaned juvenile mdx mice greatly reduces the acute onset of myofibre necrosis and dystropathology and prevents inflammation. *PLoS Curr.* 8, ecurrents.md.77be6ec30e8caf19529a00417614a072 10.1371/currents.md.77be6ec30e8caf19529a00417614a072 27679740 PMC5029885

[DMM043638C107] Terrill J. R., Pinniger G. J., Nair K. V., Grounds M. D. and Arthur P. G. (2017). Beneficial effects of high dose taurine treatment in juvenile dystrophic mdx mice are offset by growth restriction. *PLoS ONE* 12, e0187317 10.1371/journal.pone.0187317 29095865 PMC5667875

[DMM043638C108] Thangarajh M., Zhang A., Gill K., Ressom H. W., Li Z., Varghese R. S., Hoffman E. P., Nagaraju K., Hathout Y. and Boca S. M. (2019). Discovery of potential urine-accessible metabolite biomarkers associated with muscle disease and corticosteroid response in the mdx mouse model for Duchenne. *PLoS ONE* 14, e0219507 10.1371/journal.pone.0219507 31310630 PMC6634414

[DMM043638C109] Tidball J. G., Welc S. S. and Wehling-Henricks M. (2018). Immunobiology of inherited muscular dystrophies. *Compr. Physiol.* 8, 1313-1356. 10.1002/cphy.c170052 30215857 PMC7769418

[DMM043638C110] Verhaart I. E. C. and Aartsma-Rus A. (2019). Therapeutic developments for Duchenne muscular dystrophy. *Nat. Rev. Neurol.* 15, 373-386. 10.1038/s41582-019-0203-3 31147635

[DMM043638C111] Waite A., Brown S. C. and Blake D. J. (2012). The dystrophin-glycoprotein complex in brain development and disease. *Trends Neurosci.* 35, 487-496. 10.1016/j.tins.2012.04.004 22626542

[DMM043638C112] Wells D. J. (2018). Tracking progress: an update on animal models for Duchenne muscular dystrophy. *Dis. Model Mech.* 11, dmm035774 10.1242/dmm.035774 29914884 PMC6031358

[DMM043638C113] Wilson K., Faelan C., Patterson-Kane J. C., Rudmann D. G., Moore S. A., Frank D., Charleston J., Tinsley J., Young G. D. and Milici A. J. (2017). Duchenne and Becker muscular dystrophies: a review of animal models, clinical end points, and biomarker quantification. *Toxicol. Pathol.* 45, 961-976. 10.1177/0192623317734823 28974147 PMC5788182

[DMM043638C114] Winterbourn C. C. (2002). Biological reactivity and biomarkers of the neutrophil oxidant, hypochlorous acid. *Toxicology* 181-182, 223-227. 10.1016/S0300-483X(02)00286-X 12505315

[DMM043638C116] Zhao J., Yoshioka K., Miyatake M. and Miike T. (1992). Dystrophin and a dystrophin-related protein in intrafusal muscle fibers, and neuromuscular and myotendinous junctions. *Acta Neuropathol* 84, 141-146. 10.1007/BF00311386 1523969

[DMM043638C117] Zhou L. and Lu H. (2010). Targeting fibrosis in Duchenne muscular dystrophy. *J. Neuropathol. Exp. Neurol.* 69, 771-776. 10.1097/NEN.0b013e3181e9a34b 20613637 PMC2916968

[DMM043638C118] Zinellu A., Fois A. G., Sotgia S., Zinellu E., Bifulco F., Pintus G., Mangoni A. A., Carru C. and Pirina P. (2016). Plasma protein thiols: an early marker of oxidative stress in asthma and chronic obstructive pulmonary disease. *Eur. J. Clin. Invest.* 46, 181-188. 10.1111/eci.12582 26681451

[DMM043638C119] Zuo L. and Pannell B. K. (2015). Redox characterization of functioning skeletal muscle. *Front. Physiol.* 6, 338 10.3389/fphys.2015.00338 26635624 PMC4649055

